# Pre-surgery supportive and goal-oriented strategies are associated with lower post-surgery perceived distress in women diagnosed with breast cancer

**DOI:** 10.1186/s40359-021-00714-3

**Published:** 2022-01-03

**Authors:** Paolo Taurisano, Chiara Abbatantuono, Veronica Verri, Ilaria Pepe, Luigia S. Stucci, Alessandro Taurino, Marco Moschetta, Maria F. De Caro, Linda A. Antonucci

**Affiliations:** 1 Department of Basic Medical Sciences, Neuroscience and Sense Organs, University of Bari Aldo Moro, Piazza Giulio Cesare 11, 70124 Bari, Italy; 2 Breast Care Unit, Bari University Hospital, Bari, Italy; 3 Department of Education, Psychology, Communication, University of Bari Aldo Moro, Via Scipione Crisanzio 42, 70122 Bari, Italy; 4 Department of Emergency and Organ Transplantations (D.E.T.O.), Breast Care Unit, University of Bari Aldo Moro, Bari, Italy

**Keywords:** Breast cancer surgery, Distress, Coping strategies, Pre-post, Factor analysis, Multiple linear regression

## Abstract

**Background:**

Psycho-oncology literature pointed out that individual health outcomes may depend on patients’ propensity to adopt *approach* or, conversely, *avoidant* coping strategies. Nevertheless, coping factors associated with postoperative distress remain unclear, unfolding the lack of tailored procedures to help breast cancer patients manage the psychological burden of scheduled surgery. In view of this, the present study aimed at investigating: 1. pre-/post-surgery distress variations occurring among women diagnosed with breast cancer; 2. the predictivity of *approach* and *avoidant* coping strategies and factors in affecting post-surgery perceived distress.

**Methods:**

N = 150 patients (mean age = 59.37; SD =  ± 13.23) scheduled for breast cancer surgery were administered a screening protocol consisting of the Distress Thermometer (DT) and the Brief-COPE. The DT was used to monitor patients’ distress levels before and after surgery (± 7 days), whereas the Brief-COPE was adopted only preoperatively to evaluate patients’ coping responses to the forthcoming surgical intervention. Non-parametric tests allowed for the detection of pre-/post-surgery variations in patients’ perceived distress. Factor analysis involved the extraction and rotation of principal components derived from the Brief-COPE strategies. The predictivity of such coping factors was investigated through multiple regression (Backward Elimination).

**Results:**

The Wilcoxon Signed-Rank Test yielded a significant variation in DT mean scores (TW = -5,68 < -zα/2 = -1,96; p < .001) indicative of lower perceived distress following surgery. The four coping factors extracted and Varimax-rotated were, respectively: 1. *cognitive processing* (i.e., planning + acceptance + active coping + positive reframing); 2. *support provision* (i.e., instrumental + emotional support); 3. *emotion-oriented detachment* (i.e., self-blame + behavioral disengagement + humor + denial); 4. *goal-oriented detachment* (i.e., self-distraction). Among these factors, *support provision* (B = .458; β = − .174; t = − 2.03; p = .045), encompassing two approach coping strategies, and *goal-oriented detachment* (B = .446; β = − .176; t = − 2.06; p = .042), consisting of one avoidant strategy, were strongly related to post-surgery distress reduction.

**Conclusion:**

The present investigation revealed that the pre-surgery adoption of supportive and goal-oriented strategies led to postoperative distress reduction among breast cancer patients. These findings highlight the importance of timely psychosocial screening and proactive interventions in order to improve patients’ recovery and prognosis.

## Background

Breast cancer is the most frequently diagnosed cancer in women [1] and is also the major cause of cancer death in the female population (15.0%) [2]. Nevertheless, it also appears to be one of the most treatable malignancies. Indeed, the implementation of primary prevention measures, as well as early detection and intervention strategies, considerably increase the chances of survival in women developing the disease [3]. Surgery is the foremost treatment for breast cancer. However, surgical options are very different one from the other in terms of feasibility, availability, survival rate [4]. Furthermore, evidence has highlighted that breast cancer surgery—especially mastectomy—is associated with several side effects, spanning from physiological changes, such as local and regional pain syndromes, asymmetry of breast, scarring, blooding, swelling, nerve damage, to psychological negative consequences [4–6]. Indeed, in response to surgery, women may perceive the treatment they underwent (especially radical mastectomy) as painful and disfiguring, experiencing anxious preoccupation, helplessness, or hopelessness, besides positive coping strategies aimed at ‘fighting’ or ‘redefining’ the problem [7]. Accordingly, a recent investigation pointed out that the management of breast cancer surgery is associated with several psychosocial factors (e.g., attitudes and representations towards the disease; self-esteem; coping strategies, including emotional and social support) [8]. Further studies revealed that, besides needs of physical assistance before and after surgery, patients’ need for psychosocial care is often neglected for lack of data and tailored procedures [9]. This calls research and clinical practice for putting more efforts in identifying risk and protective factors that may soften the burden associated with diagnosis, surgery, and recovery, in view of the association between breast cancer and psychiatric disorders (e.g., sleep disorders; fatigue; combined anxiety and depressive symptoms) [10, 11].

Across all the aforementioned factors, it has been shown that both diagnostic and intervention processes, especially when surgery is involved, entail a significant amount of stress [12, 13]. Stress has been defined as ‘a non-specific response of the body to any demand for change’ [14]. Consolidated views have endorsed the distinction between distress and eustress as they represent, respectively, non-adaptive and adaptive responses to the stimuli causing stress [15, 16].

On the one hand, it has been largely reported both diagnosis and treatment for breast cancer may represent disruptive events eliciting distress [17], with higher responses to fear of cancer recurrence, surgery-related pain and concerns, and uncertainty about the future as core manifestations [4, 6, 12, 18]. On the other hand, moderate exposure to stress could play a crucial role in fostering psychological resilience in cancer patients [17, 19], as it may prevent the onset or reduce the effects of illness-related intrusive thoughts, thus enhancing recovery [12, 19]. This interplay between distress and eustress in patients with cancer shows a high inter-individual variability [20] which mainly relies on subjective differences between patients in their coping approaches [21].

Coping has been defined as a multidimensional construct covering a variety of everchanging efforts (thoughts or behaviors) to meet the demands of a stressful situation [22]. An extensive body of literature relates Lazarus & Folkman’s distinction [22] between *emotion-focused* coping (e.g., distraction; thought suppression; lower expectations), involving the struggle to reduce the arousal, and *problem-focused* coping (e.g., cognitive and emotional acceptance; improving relationships), which is instead supposed to remove the stressor at source [23]. Further contributions [24–26] discriminate between *approach* and *avoidant* coping strategies, whether they be situational or dispositional coping responses [27, 28]. While approach coping mainly consists of adaptive strategies that people actively adopt in response to the stressor, avoidant coping represents an attempt to ignore or take distance from the stressful situation [24, 26].

According to the complexity of the aforementioned distress-eustress interplay associated with the management of breast cancer surgery, it has been recently reported that post-traumatic cancer experiences might promote adaptive coping resources [29, 30] as they can drive a set of cognitive and behavioral attempts (i.e., active coping) aimed at managing the disease and the postoperative phase. Notably, positive coping (e.g., positive thinking, acceptance, spiritual and social support, and role retention) has been shown to contribute significantly to the personal adjustment related to the experience of the illness itself. Furthermore, it has been associated with increases in the overall quality of life even in case of cancer-related problems and complications [20]. However, while some patients may attempt and succeed in adapting to stress through positive coping, others may opt for negative coping strategies, such as avoidance and disengagement, although these responses seem to be maladaptive and less effective than the positive ones [31].

Similar to distress [32, 33], coping strategies seem to evolve over time [13, 22] as they reflect patients’ psychological status [34, 35]. Indeed, a recently proposed view shows that, while survival fosters positive cognitive restructuring as a lifetime adaptive strategy [13], adverse effects of surgery may have a negative impact on health-related quality of life [36], representing challenging stress factors to cope with.

Based upon the literature reported, the present study aims at investigating: 1. distress variations occurring at pre-/post-surgery among women diagnosed with breast cancer; 2. the relationship between the types of coping strategies and factors adopted preoperatively by patients, and perceived post-surgery distress. Thus, we expect to find different distress levels before and after surgery, based on patients’ coping adjustments used to tackle the psychological load of undergoing such invasive yet necessary procedures. These adjustments may be interpreted as *approach* or *avoidant* coping strategies [24, 25], associated with patients’ manifold stress responses.

## Methods

### Study design and sample determination

The pre-post observational study involved the participation of women diagnosed with breast cancer while hospitalized to undergo primary surgery. To this end, data were collected from January 2019 to March 2020 within the Breast Care Unit (BCU) – Department of General Surgery “N. Balestrazzi” based at Bari University Hospital (Italy). Assessment procedures involved the administration of two psychological screening inventories at different times. Prior to the assessment, both socio-anamnestic and clinical data and information (i.e., initial diagnosis or relapse; presence or absence of metastatic lesions; first-degree familial vulnerability for any cancer disease; type of surgery (i.e., mastectomy vs. quadrantectomy) were obtained by trained psychologists belonging to the BCU multidisciplinary team. To be eligible for the study, patients would not have met the following exclusion criteria: age under 18 years; past psychiatric history assessed through the *Structural Clinical Interview for DSM-IV* [37]; past substance abuse history; current alcohol or drug use; diagnosed medical comorbidities (based on medical records).

### Psychological assessments

In order to evaluate and monitor stress levels in oncology patients, the Distress Thermometer (DT) [38–40] was adopted, consistent with the National Comprehensive Cancer Network (NCCN) Guidelines® [41, 42]. This screening tool consists of a visual-analog scale used to screen for patients’ distress status through a summary score ranging from 0 (meaning ‘no distress’) to 10 (meaning ‘extreme distress’). The single item that forms the scale refers to the week preceding the assessment. DT scores higher than 4 may indicate clinically significant values corresponding to moderate (DT scores between 4 and 7) or severe (DT scores > 7) psychological distress [43, 44]. The Italian version of this screening tool (cut-off = 4) has been showing good psychometric properties [45]. The DT was administered before tumor removal (i.e., when participants were admitted to the hospital) and a week after surgery.

The Brief-COPE [46–48] was used for the detection of the most common coping strategies used by patients to manage surgery-related distress. This 4-point Likert scale (from 1 = ‘I haven’t been doing this at all’ to 4 = ‘I have been doing this a lot’) is composed of 28 items referring to 14 distinct strategies, i.e., 1. self-distraction, 2. active coping, 3. denial, 4. substance use, 5. use of emotional support, 6. use of instrumental support, 7. behavioral disengagement, 8. venting, 9. positive reframing, 10. planning, 11. humor, 12. acceptance, 13. religion, and 14. self-blame. The Brief-COPE was designed to reduce the length of the original Coping Orientation for Problems Experienced (COPE) [25], representing one of the less time-consuming instruments for coping assessment [49] grounded on Lazarus and Folkman’s stress model [22]. Contrary to the original inventory, this abbreviated version refers to a ‘more specific’ situation deemed to be stressful. Given the purposes and the design of the present study, no threshold was considered to evaluate the occurrence of each coping strategy. The Italian version of the Brief-COPE showed acceptable reliability and highlighted the usefulness of this measure to screen for coping responses in relation to specific events, as reported in Monzani et al. [28]. The Brief-COPE was administered only before surgery.

### Statistical analysis

All statistical analyses were performed by means of the 20th version of the *Statistical Package for Social Science* (SPSS 20.0) [50].

### Investigations on demographic and psychological variables

Frequency, mean, and standard deviation were used to describe the sample demographic and clinical characteristics (i.e., age; educational level; marital status; children; familiarity with cancer; type of surgery). The distribution of DT preoperative mean scores was investigated through the Kolmogorov–Smirnov Test. For our sample, for both timepoints, data did not fit a normal distribution. Hence, the non-parametric Wilcoxon Signed-Rank Test was used to compare repeated measurements (i.e., the main effect of time on self-reported distress).

### Generation of coping strategy factors

In order to detect the types of coping strategies suitable for factor analysis as well as for predictive analysis, pre-surgery coping strategies assessed through the Brief-COPE were interpreted based on Roth’s [24] and Carver’s [46] bidimensional model, as previously reported in literature [26, 35]. Consistently, the scores concerning patients’ strategies to deal with pre-surgery distress were categorized as approach or avoidant coping strategies. The principal component analysis with Varimax rotation enabled the extraction of the most significant factors from the original 14 coping strategies. Hereby, principal components emerging from this analysis will be named as “coping strategy factors”.

To investigate whether our derived coping factors could be associated with demographic and clinical aspects, we have performed correlation analysis between patients’ background variables, both demographic (i.e., age, education, employment, marital status, children) and disease-related (i.e., cancer familiarity, and type of surgery), and our derived coping strategy factors. To this end, Pearson’s correlation coefficient was used to analyze continuous variables and Spearman’s correlation for ordinal variables. Pillai’s trace or Mann–Whitney U test were also performed with categorical variables, considering the small-sized sample and the non-normal distribution of data. To reduce the probability of Type I error, a Benjamini–Hochberg False Discovery Rate (FDR) adjustment [51] was applied to the error level. P values were therefore < 0.05, FDR corrected, and resumed in Table 7.

As a last step, we wanted to investigate whether and how the derived coping strategies factors could predict post-surgery distress levels. Thus, the generated coping strategy factors, together with patients’ age at surgery and the type of surgical intervention, entered as predictors a multiple linear regression (Backward Elimination model) aimed at predicting post-surgery DT scores. Evidence of significance from Bartlett's Sphericity Test, along with an acceptable Kaiser-Meyer Olkin (KMO) index, ensured the reliability of the correlation matrices and allowed for the extraction of the four main factors with Varimax rotation. P-values lower than 0.05 were interpreted as statistically significant.

## Results

### Investigations on demographic and psychological variables

A total of 150 consecutive in-patients diagnosed with breast cancer (28 of which dealing with tumor relapse) and candidate for surgery were recruited and took part in the present study. Table 1 shows the demographic and clinical characteristics of the participants in the study sample. All participants were women aged approximately 59.37 years (mean; SD = 13.23; range 28–86) and met the pre-determined eligibility criteria. Indeed, no participant was diagnosed with any mental or further physical disease, presenting instead sufficiently good health conditions to minimize the risks associated with surgery. Descriptive analyses of socio-demographic data revealed a predominance of women graduated from secondary school (28.2%), employed (62.6%), married (74.3%), and with two children on average. More than half of the sample reported familiarity with breast cancer (57.3%). Considering the clinical history collected from each participant, only one patient suffered from metastatic disease, having been diagnosed with lung metastases, whereas the remaining 149 subjects had non-metastatic carcinoma. All participants underwent the scheduled surgical intervention and pre-post psychological assessment. Regarding the type of surgery, 34.4% of them had a total mastectomy, whereas the remaining 65.6% underwent less invasive quadrantectomy.

**Table 1:** Demographic and clinical characteristics of the study sample (n = 150)

Variable	
*Age*	
Mean (SD)	59.37 (13.23)
Range	28–86
*Educational level* (missing system: 1)	
Primary school	31 (20.8)
Middle school	42 (28.2)
Secondary school	42 (28.2)
University degree	34 (22.8)
*Employment status*	
Not employed	55 (36.7)
Employed	95 (63.3)
*Marital status* (missing system: 2)	
Single	13 (8.8)
Married	110 (74.3)
Separated/divorced	8 (5.4)
Widowed	17 (11.5)
*Children*	
Mean (SD)	1.84 (1.01)
Range	0–4
*Familiarity* (missing system: 1)	
No	63 (42.3)
Yes	86 (57.7)
*Type of surgery* (missing system: 19)	
Mastectomy	45 (34.4)
Quadrantectomy	86 (65.6)

DT pre-surgery mean score was equal to 6.63 (SD = 2.60). Based on cut-off criteria, 69 women (46% of the sample) reported scores between 4 and 7, indicating the occurrence of moderate distress. On the other hand, 61 women (40.7%) showed distress levels higher than 7, meaning that they were experiencing severe clinical distress, as shown in Table 2. DT mean score dropped to 5.38 (SD = 2.49) after surgery. Indeed, this phase was characterized by severe levels of distress reported by 22.7% of patients (Table 2). Further comparison between pre- and post-surgery DT scores confirmed a significant distress reduction (TW = − 5.68 < − zα/2 = − 1.96; p < 0.001) following surgical procedures (Table 3).

**Table 2:** Characteristics of the Distress Thermometer (DT) variable measured before and after surgery

	Pre-surgery	Post-surgery
*DT score*		
Mean (SD)	6.63 (2.60)	5.38 (2.49)
Range	0–10	0–10
*DT level*		
Absent	5 (3.3)	9 (6.0)
Mild	15 (10.0)	19 (12.7)
Moderate	69 (46.0)	88 (58.7)
Severe	61 (40.7)	34 (22.7)

**Table 3:** Variations between pre- and post-surgery Distress Thermometer (DT) scores resulting from Wilcoxon Signed-Rank Test

Wilcoxon Signed-Rank Test	Values
N	150
Test statistic	738.50
Standard Error	263.00
Standardized Test Statistic	− 5.681
Asymptotic Sig. (2-sided test)	< .001

Regarding coping strategies, a mean score was reckoned for each Brief-COPE domain (Table 4). In particular, the highest scores pertained to the so-called *approach coping* strategies, i.e., acceptance (mean = 5.65; SD = 1.98), positive reframing (mean = 5.30; SD = 2.08), use of emotional support (mean = 4.97; SD = 1.83), planning (mean = 4.95; SD = 2.15), and use of instrumental support (mean = 4.63; SD = 1.87). Conversely, the lowest scores concerned substance use (mean = 2.06; SD = 0.59), behavioral disengagement (mean = 2.54; SD = 1.38), and self-blame (mean = 2.99; SD = 1.66).

**Table 4:** Brief-COPE mean scores in breast cancer patients

Variable	Mean ± SD
Self-distraction	5.34 ± 1.92
Active coping	3.75 ± 2.02
Denial	3.07 ± 1.76
Substance use	2.06 ± .59
Emotional support	4.97 ± 1.83
Instrumental support	4.63 ± 1.87
Behavioral disengagement	2.54 ± 1.38
Venting	4.36 ± 1.91
Positive reframing	5.30 ± 2.08
Planning	4.95 ± 2.15
Humor	3.03 ± 1.75
Acceptance	5.65 ± 1.98
Religion	5.24 ± 2.15
Self-blame	2.99 ± 1.66

### Generation of coping strategy factors

Factor analysis allowed for the extraction and rotation of four factors distinguishable by their type of functioning (*approach* vs. *avoidant* coping). The variables scrutinized were the 14 strategies theoretically identified by the Brief-COPE. Before proceeding with factor extraction, sampling adequacy and appropriateness of the correlation matrix of the factorable variables were tested, respectively, through Kaiser-Meyer Olkin (KMO) Test and Bartlett’s Test for Sphericity. A KMO index equal to 0.72 and the significance (p-value < 0.001) resulting from Bartlett’s Test (= 598.86) confirmed the psychometric adequacy for factor analysis, allowing for the 14 variables to be grouped in summary components (i.e., “coping strategy factors”).

In particular, four coping strategy factors with an eigenvalue higher than 1.0 were detected by using the Varimax orthogonal rotation. Overall, these factors implied 58.63% of the total variance of the correlation matrix. The absolute values of the highest factorial weights were highlighted. The eigenvalue graph confirmed a flattening of the curve starting from the fifth factor (Table 5).

**Table 5:** Variance explained by both unrotated and Varimax-rotated factors (extraction method: Principal Component Analysis)

Coping strategy factor	Initial eigenvalues			Extraction sums of squared loadings			Rotation sums of squared loadings		
	Total	% of variance	Cumulative %	Total	% of variance	Cumulative %	Total	% of variance	Cumulative %
1_ Cognitive processing	3.782	27.015	27.015	3.782	27.015	27.015	2.397	17.122	17.122
2_Support provision	1.785	12.753	39.768	1.785	12.753	39.768	2.387	17.050	34.172
3_Emotion-oriented detachment	1.533	10.953	50.721	1.533	10.953	50.721	2.188	15.628	49.799
4_Goal-oriented detachment	1.108	7.916	58.637	1.108	7.916	58.637	1.237	8.837	58.637
5	1.033	7.377	66.013						
6	.955	6.819	72.832						
7	.764	5.458	78.290						
8	.654	4.675	82.965						
9	.589	4.207	87.172						
10	.498	3.558	90.730						
11	.438	3.125	93.855						
12	.392	2.803	96.658						
13	.260	1.858	98.516						
14	.208	1.484	100.000						

The values that indicate the strength of the relationship between the variables (i.e., coping strategies) and the four factors are summarily reported in the correlation matrix (Table 6).

**Table 6:** Pattern coefficients for 14 coping strategies (i.e., Brief-COPE scores) after Varimax Rotation with Kaiser Normalization

Coping strategy	Coping factor			
	Cognitive processing	Support provision	Emotion-oriented detachment	Goal-oriented detachment
Venting	− .070	.651	.200	.071
Instrumental support	.244	.830	.099	− .009
Emotional support	.242	.833	.012	.011
Self-distraction	.236	.279	.194	.701
Denial	− .179	.200	.582	.228
Humor	.273	.053	.635	.037
Behavioral disengagement	− .266	.337	.613	.239
Substance use	.118	.039	.531	− .038
Positive reframing	.639	− .010	.413	− .290
Acceptance	.740	.036	.113	− .115
Active coping	.657	.278	.099	.375
Planning	.765	.345	− .061	.291
Religion	.150	.383	.010	− .553
Self-blame	.178	− .014	.710	− .016

The first coping strategy factor (i.e., *cognitive processing*) absorbed 17% of the overall variance, and was positively correlated with some adaptive strategies, i.e., planning, acceptance, active coping, and positive reframing. The same applied to the second factor (i.e., *support provision*), having a positive correlation with the use of instrumental and emotional support, respectively. In contrast, a correlation between maladaptive coping strategies and the remaining factors was observed. In particular, the third factor (i.e., *emotion-oriented detachment*) was related to self-blame, behavioral disengagement, humor, and denial, whereas the fourth factor (i.e., *goal-oriented detachment*) showed a positive correlation with self-distraction, and a negative correlation with religion.

### Association between coping strategy factors and clinical and demographic variables

Bivariate associations between clinical and demographical variables and the four coping strategy factors are displayed in Table 7. No statistically significant association was found between the four coping factors and all the clinical and demographic variables investigated (all p > 0.05, FDR corrected).

**Table 7:** Associations between coping and background variables (Sig. = Significance)

		Coping factor			
		Cognitive processing	Support provision	Emotion-oriented detachment	Goal-oriented detachment
*Age*	Pearson Correlation	− .02	− .01	.02	− .18
	Sig. (2-tailed)	.91	.92	.91	.31
*Educational level*	Spearman Coefficient	− .05	− .09	− .08	.23
	Sig. (2-tailed)	.80	.53	.60	.10
*Employment status*					
Not employed	Mean (n = 55)	− .05	.07	.12	− .21
Employed	Mean (n = 95)	.03	− .04	− .07	.12
	Mann–Whitney test standardized	.21	− .64	− .93	1.88
	p-value	.91	.80	.61	.33
*Marital status*					
Single	Mean (n = 13)	− .01	.04	.05	.05
Married	Mean (n = 110)	.11	− .62	− .60	− .25
Separated/divorced	Mean (n = 8)	.47	.23	.73	− .12
Widowed	Mean (n = 17)	− .11	.08	− .12	− .11
	Pillai’s Trace	.13			
	p-value	.33			
*Children*	Spearman Coefficient	.09	.01	.14	− .02
	Sig. (2-tailed)	.53	.92	.33	.91
*Familiarity*					
No	Mean (n = 63)	− .18	− .11	− .10	.01
Yes	Mean (n = 86)	.11	.08	.07	− .01
	Mann–Whitney test standardized	1.59	1.26	1.30	− .27
	p-value	.33	.47	.47	.91
*Type of surgery*					
Mastectomy_1	Mean (n = 45)	.22	.17	.03	.21
Quadrantectomy_0	Mean (n = 86)	− .07	− .04	.01	− .08
	Mann–Whitney test standardized	− 1.56	− 1.60	− .23	− 1.62
	p-value	.33	.33	.91	.33

### Determinants of post-surgical distress levels

The relationship between the four coping factors, patients’ age, surgical procedure (Quadrantectomy_0; Mastectomy_1), and the reduction in post-surgery distress was analyzed through multiple linear regression (Table 8). The final model, resulting from the application of the Backward Elimination technique, highlighted the statistical significance of both *support provision* and *goal-oriented detachment* in predicting post-surgical distress variation. Both predictors were in an inversely proportional relationship with distress variation (B [Unstandardized coefficient] = − 0.458; β [Standardized coefficient] =  − 0.174; t = − 2.03; p = 0.045; (B = − 0.446; β = − 0.176; t = − 2.06; p = 0.042)). Conversely, *cognitive processing* (t = − 0.78; p = 0.43) and *emotion-oriented detachment* (t = 0.80; p = 0.43) were not found to be significant in reducing post-surgery distress.

**Table 8:** Multiple Linear Regression aimed at predicting post-surgery distress levels based on the coping strategy factors, age, and type of surgery

Model	Unstandardized coefficients		Standardized coefficients	t	Sig.	95.0% Confidence Interval for B	
	B	Standard error	Beta			Lower bound	Upper bound
(Constant)	− 1.515	1.105		− 1.371	.173	− 3.703	.672
Cognitive processing	− .176	.225	− .069	− .783	.435	− .621	.269
Support provision	− .484	.229	− .184	− 2.112	.037	− .938	− .031
Emotion-oriented detachment	.196	.245	.070	.801	.425	− .288	.680
Goal-oriented detachment	− .459	.224	− .181	− 2.044	.043	− .903	− .014
Age	.002	.018	.010	.109	.914	− .033	.037
Quadrantectomy_0/Mastectomy _1	.371	.487	.068	.762	.448	− .593	1.336
(Constant)	− 1.276	.223		− 5.730	.000	− 1.717	− .836
Support provision	− .458	.226	− .174	− 2.027	.045	− .904	− .011
Goal-oriented detachment	− .446	.217	-.176	− 2.057	.042	− .875	− .017

B = unstandardized coefficient; Beta = standardized coefficient; Sig. = significance

Patients’ age and surgical procedures were not allowed to enter the final regression model (all p > 0.05), thus showing no association with distress variation.

## Discussion

The present study sought to determine whether and to which extent transition from pre- to post-surgery could lead to a distress variation among women affected by breast cancer. More specifically, based on a longitudinal design, the research also focused on the predictive role played by diverse coping strategies and related factors in reducing post-surgery distress.

The first research aim has been achieved through the comparison of DT mean scores reported at different times, i.e., before and after surgery. The non-parametric tests performed revealed significant differences in participants’ scores between the two measurements (p < 0.001), indicating a distress reduction following surgery. An explanation for such drop in the perceived psychological burden may lie in patients’ repertoire of cognitive and behavioral adjustments aimed at managing the uncertainty and fear of undergoing invasive medical procedures. Indeed, tumor resection seems to entail a notable amount of distress associated with the fear of dying [52, 53], besides the fear of cancer recurrence [54], loss of control over bodily integrity [18], and surgical complications [4, 55]. The sudden situation changes concerning cancer diagnosis and treatment may provoke symptoms of emotional distress (e.g., self-criticalness and hopelessness) that are associated with lower medical compliance and lack of existential support [54], with potential psychological implications following surgery. Notably, surgery may also shape patients’ quality of life, resulting in a more complicated physical and psychological recovery, especially for mastectomized patients [5, 7]. Accordingly, mastectomy is often associated with several adverse effects (e.g., intense pain in the arm and breast; phantom breast syndrome; anatomic changes of shoulder girdle; motor impairment), limitations in daily living and professional activities, physical changes perceived as mutilation, disfigurement or loss of femininity [4, 7]. Increased distress and side effects also pertain to breast-conserving surgery (including quadrantectomy) that is associated with asymmetry, pain, and further risks and effects deriving from adjuvant therapy [4]. However, acute distress elicited by surgery is also expected to enact adaptive responses such as focusing on new possibilities for life rather than brooding on cancer recurrences, wishful thinking about the near future, and/or effectively processing positive and negative aspects regarding their actual health condition [7, 8, 12, 54]. Given the multidimensional nature of distress responses, interindividual differences may depend on patients’ resources which include, among all, patterns of beliefs [56], perceptions of cancer treatment [54], and/or locus of control [52]. Considering these speculations, future studies are warranted to investigate cognitive and emotional mediation and moderation factors of the observed pre-post surgery distress variation.

As coping embraces both cognitive and behavioral responses that could be labeled as *approach* or *avoidant* strategies towards stressful experiences, the second research aim has been achieved through factor extraction and rotation, and further predictive analyses through the Backward Elimination Regression. In line with a recent study that has shed light on the relationships that may link cancer-related distress, coping, and quality of life [35], the present study has detected specific factors that might have contributed to the significant distress reduction emerging over the brief timespan of in-patient surgery. As a result, half of the factors extracted have been shown to be positively associated with approach coping strategies. In particular, *cognitive processing* (i.e., the factor with the largest eigenvalue) was related to planning, acceptance, active coping, and positive reframing, whereas *support provision* covered instrumental and emotional support, related to each other, and thus loading on a single factor as reported in Monzani et al. [28]. The remaining two avoidant factors were *emotion-oriented detachment* (i.e., self-blame; behavioral disengagement, humor, denial), primarily aimed at avoiding emotional overwhelm [20, 57], and *goal-oriented detachment* (i.e., self-distraction), resulting in more practical attempts to manage distress (also confirmed by the negative correlation with the *religion* variable) that may prevent role weakening [20, 58].

According to our findings, among the 14 Brief-COPE variables, *humor* has emerged as an avoidant response to preoperative distress. This result could depend on item ambiguity [26], sample characteristics, and theoretical models adopted [23]. *Humor* has indeed been defined as a relatively unstable variable that might fall under emotion-focused strategies [23], loading on either approach or avoidant factors [26].

Besides factorial findings, the novelty of our study concerns the predictive value of three coping strategies, loading on two coping strategy factors, that led to a drop in post-surgery DT scores. Regression findings indicate the strongest factors in lowering patients’ self-reported distress are *support provision*, encompassing both instrumental and emotional support (i.e., approach coping strategies), and *goal-oriented detachment*, consisting of self-distraction (i.e., avoidant coping strategy). Patients who perceived being supported and committed to functional activities before undergoing surgery had greater chances to show mild to moderate distress as well as lower chances to experience severe distress following surgery. This result could be explained in view of the key role played by such proactive behaviors in enhancing well-being and resilience among cancer patients undergoing surgery [35]. Firstly, seeking and obtaining comfort, understanding, information and/or advice may act as adaptive strategies, resulting in a more effective and tangible way to rapidly relieve distressed patients, compared with cognitive approach coping implying the reappraisal of breast cancer diagnosis and treatment [59]. In fact, the mental efforts involved in *cognitive processing* likely require more time for the patient alone to reframe and accept surgical experiences, and thus to perceive a significant distress reduction. Secondly, focusing on leisure and work emerges as a practical attempt adopted by patients to take back their occupational role during early survivorship [60]. Such goal-directed behaviors are proven to be linked with self-efficacy [61], and to constitute positive changes in important life priorities and roles [30] that are coherent with patients’ perceived social support [58].

Besides background variables, the remaining two coping factors extracted (i.e., *cognitive processing* and *emotion-oriented detachment*) were not found to be associated with post-surgery distress. It follows that avoidant coping strategies and factors may be interpreted as either adaptive or maladaptive on individual basis as they can also contribute to fostering short-term adjustments to surgery. Indeed, combined approach-avoidant coping consisting of both supportive and goal-oriented strategies appears to be more effective in lowering surgery-related distress, consistent with recent studies claiming that breast cancer patients can feel unburdened through self-regulation and help from others [58, 62]. However, extensive use of avoidant strategies could also reveal the tendency to focus on external circumstances beyond one’s perceived control, entailing an increased risk for developing mental health issues over time [63]. As these coping responses have been found significant in mediating locus of control and distress vulnerability [63], paying clinical awareness to these mechanisms while encouraging the timely use of behavioral coping strategies might lead to notable improvements in patients’ post-surgery prognosis.

### Limitations

Despite the overall reliability of the predictive analyses performed, our study has some limitations that require future studies and validations to be overcome. One of the major limitations is represented by the shortness of the assessment protocol administered. Indeed it should be also noted that, differently from previous literature [7, 20, 64, 65], our results highlighted that patients’ age and the type of surgery could predict neither growth, nor drop, in patients’ perceived distress. This difference could be explained in light of the brief timespan occurring between pre- and post-surgery assessments in our study. Indeed, the whole study lasted one week, from patients’ admission to patient’s discharge). We may speculate that this time constraint did not allow us to fully investigate the surgical experience from a truly longitudinal (i.e., not “pre-post”) perspective, thus also not allowing us to investigate the mid- and long-term sequelae that likely require more time to arise [4], and in which specific post-treatment side effects may potentially play a more important role. Future studies are warranted to investigate this speculation.

Another weakness is represented by the administered self-report questionnaires. Indeed, albeit time-effective and recommended to screen for patients’ situational responses [26, 35, 41], the DT and Brief-COPE may be threatened by measurement errors such as respondent biases (e.g., social desirability, poor introspective ability, misreporting risks while asking ‘sensitive’ questions). Consistently, it has been shown that the DT instrument measures non-specific psychological distress compared to psycho-oncology gold standards that allow for the discrimination between surgically driven and general distress in breast cancer patients [66, 67]. Furthermore, consistent with previous literature, the sole administration of the DT not coupled with the Problem List (i.e., an additional instrument that might help patients to clearly identify sources of their distress [42]) may only deliver a partial picture of the individual distress level. A further limitation of the current study is that our data collection did not include other potentially relevant psychological aspects of the breast cancer experience. Aspects that were not object of this study but that still need to be explored include, for example, patients’ mood and medium or long-term psychological correlates of surgical procedures (e.g., body perception, post-surgery coping strategies, dispositional coping styles, and locus of control).

Based on all the listed limitations, we believe our findings need to be externally validated and further extended through the collection of additional clinical variables, the administration of more extensive psychological assessments, and a broader longitudinal timeframe. These methodological strategies could allow for extended and reliable monitoring of coping, distress, and other psychological correlates of breast cancer surgery at multiple time points. Thus, future studies are warranted to further investigate the validity of our findings in cohorts assessed with different psychological assessments and in a more longitudinal (i.e., not just “pre/post”) framework.

## Conclusions

Overall, this study has provided preliminary evidence about the associations between patients’ distress variations and coping repertoire, demonstrating the predictive and preventive function of both *support provision* and *goal-oriented detachment* before undergoing breast cancer surgery. These results endorse the importance of fostering personalized psychosocial intervention, as the acquisition, development, and/or promotion of supportive and goal-directed strategies may improve patients’ recovery through a significant distress reduction. From a biopsychosocial perspective, it is indeed necessary to screen for psychological distress pre- and post-operatively, considering the high psychological load breast cancer patients usually manage while hospitalized. In addition, addressing distress and coping resources through person-friendly tools and methods could be helpful not only in bridging the gap between professionals and patients, but also in disambiguating coping operative definitions (e.g., *humor*) and patients’ cancer representations.

We believe that, if further externally validated, our results may have intriguing implications for breast cancer clinical practice. Indeed, enhancing patients’ ability to benefit from social support and role strengthening may be advisable to minimize the risk of experiencing severe distress after surgery, as these coping strategies constitute protective factors for dealing with such invasive yet needful treatments over the short-term period.

For example, in clinical contexts, individual distress responses to surgical treatment may be reported by psychologists during scheduled team briefings with medical staff (i.e., before surgery and one week after surgery) to achieve a shared understanding of patients’ distress management and thus inform local guidelines for healthcare professionals. This way, medical and nursing staff working in multidisciplinary teams could be enriched with such information regarding the psychological dynamics of the surgical process, and provide a better, more tailored and personalized assistance, supporting them from a comprehensive biopsychosocial perspective, and strongly taking into account the intra- and inter-individual susceptibility to surgical distress.

## Data Availability

The datasets used and/or analysed during the current study are available from the corresponding author on reasonable request.

## Abbreviations

BCU: Breast Care Unit
Brief-COPE: Abbreviated version of the Coping Orientation for Problems Experienced
DSM: Diagnostic and statistical manual of mental disorders
DT: Distress thermometer
FDR: False discovery rate
KMO: Kaiser–Meyer–Olkin
